# Draft Genome Sequence of Citrobacter cronae Awk (Sequence Type 880), Associated with Edible Snails Commercially Available in Awka, Nigeria

**DOI:** 10.1128/mra.00634-22

**Published:** 2022-09-22

**Authors:** Arthur C. Okafor, Adriana Cabal Rosel, Frank C. Ogbo, Anna Stöger, Franz Allerberger, Werner Ruppitsch

**Affiliations:** a Institute of Medical Microbiology and Hygiene, Austrian Agency for Health and Food Safety, Vienna, Austria; b Department of Applied Microbiology and Brewing, Nnamdi Azikiwe University, Awka, Nigeria; Loyola University Chicago

## Abstract

We describe the draft genome sequence and annotation of Citrobacter cronae strain Awk (sequence type 880), recovered from fresh edible snails (Achatina achatina) commercially available in Awka Metropolis, Nigeria. The genome contains 4,629 protein-coding genes, 107 RNA-coding genes, and several antimicrobial resistance genes, including *bla*_CMY-98_ and *qnrB12*.

## ANNOUNCEMENT

*Citrobacter* species are usually present in soil, water, food, and the intestines of food animals, such as snails. They are among the *Enterobacteriaceae* members of rising clinical importance (1–3). Citrobacter cronae was first described in 2020, following its isolation from rectal swabs of immunocompromised patients in Germany (1). Antimicrobial-resistant (AR) strains of *C. cronae* have been isolated from fennec fox (Vulpes zerda) imported into China from Sudan and from termites and fruits in Senegal (4, 5).

*C. cronae* sequence type 880 (ST880) strain Awk was isolated in September 2021 from fresh snails (Achatina achatina) commercially available at Eke Awka Market in Awka Metropolis, Nigeria. Samples were processed as described previously (6), and the strain was identified as *Citrobacter* using standard bacteriological methods (3). The colonies were subcultured on blood agar (bioMérieux, Marcy-l’Étoile, France) under aerobic conditions for 24 h at 35°C and identified using matrix-assisted laser desorption ionization–time of flight mass spectrometry (MALDI-TOF MS) (Bruker, Bremen, Germany) with Biotyper v4.1.100 software. Genomic DNA was extracted from the colonies using the MagAttract high-molecular-weight (HMW) DNA kit 48 (Qiagen, Hilden, Germany), and its concentration was measured using the DropSense 16 system (Trinean NV, Gentbrugge, Belgium). Genomic libraries were prepared using the Nextera XT DNA library preparation kit (Illumina, San Diego, USA) and sequenced (paired-end, 2 × 300-bp format) using the Illumina MiSeq instrument. *De novo* assembly of the raw reads was performed using SPAdes v3.15.2 (https://cab.spbu.ru/software/spades/), and the assembly was assessed for quality using FastQC v0.11.7 and Trimmomatic v0.36. The NCBI Prokaryotic Genome Annotation Pipeline v6.1 was used for annotation (7). Whole-genome-based taxonomic analysis was conducted using digital DNA-DNA hybridization (dDDH) (https://tygs.dsmz.de/), and the multilocus sequence typing (MLST) scheme was also engaged. An *ad hoc* core genome MLST (cgMLST) scheme consisting of 3,487 targets was constructed using SeqSphere+ v8.3.1 (Ridom, Münster, Germany) for comparison with all *C. cronae* genomes available at NCBI GenBank as of 17 June 2022. The strain was further characterized using the Comprehensive Antibiotic Resistance Database (https://card.mcmaster.ca/analyze/rgi) (8) and MobileElementFinder (https://cge.food.dtu.dk/services/MobileElementFinder/) (9). All software was used with default parameters.

**FIG 1:**
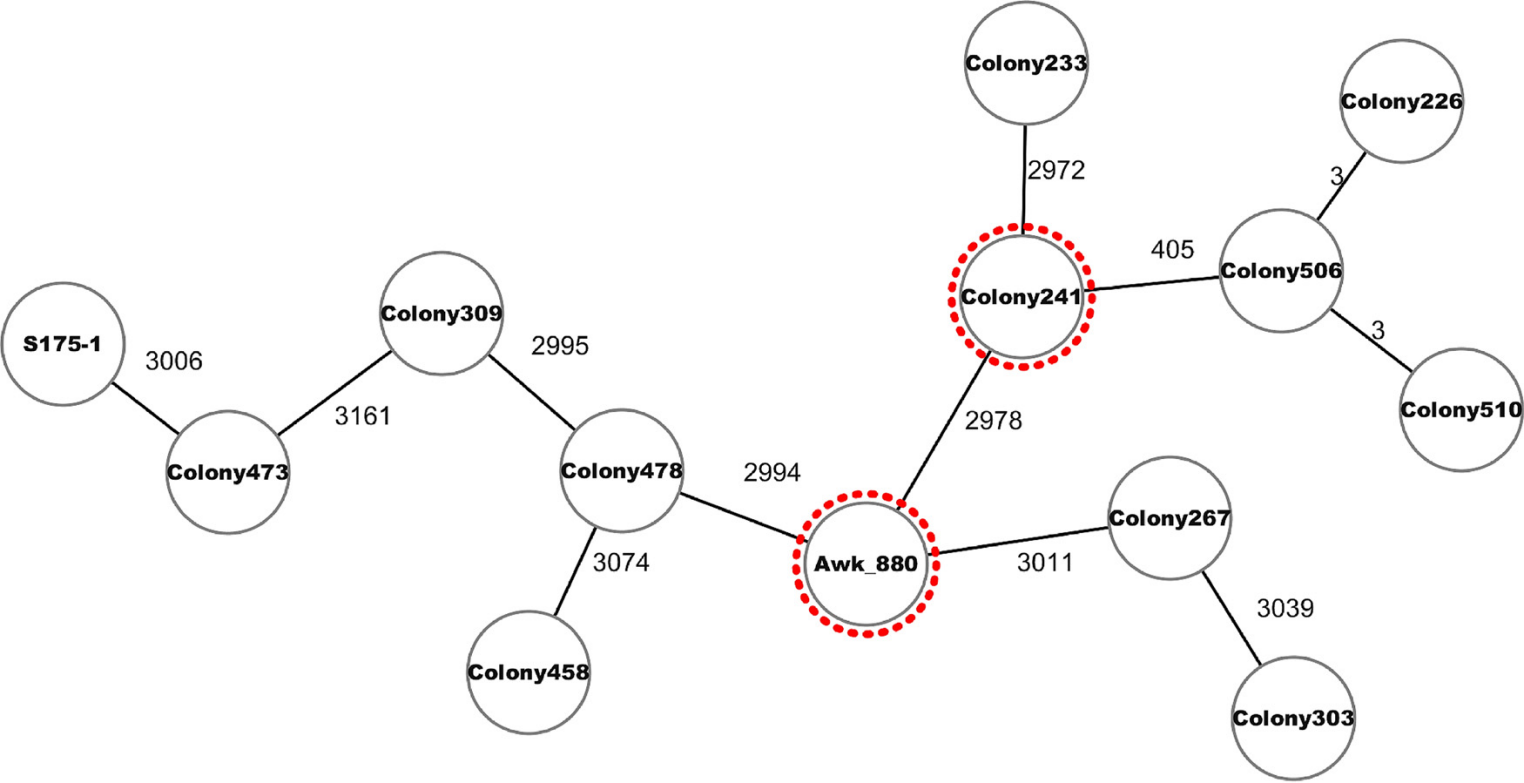
Minimum spanning tree generated from the cgMLST comparison of strain Awk ST880 with all other *C. cronae* genomes at NCBI GenBank. The numbers next to the lines represent allelic differences.

Using MALDI-TOF MS and dDDH, strain Awk was identified as Citrobacter freundii and *C. cronae*, respectively. The genome of strain Awk consists of 5,021,748 bp with 76-fold average coverage and 52.1% GC content. The average number of reads and read length were 1,940,552 and 197, respectively. There were 116 contigs, and the *N*_50_ value was 125,110 bp. Genome annotation predicted 4,835 genes, 4,629 protein-coding genes, 76 tRNAs, 31 rRNAs, 13 noncoding RNAs, and 86 pseudogenes. MLST revealed a new sequence type for strain Awk and assigned ST880. The cgMLST comparison with all genomes in NCBI GenBank (Fig. 1) showed that the closest relative was a strain of *C. cronae* (Colony 241) isolated from food in Thailand (GenBank accession number CP069768.1), with 2,978 allelic differences. Moreover, our strain contained several AR genes, including *bla*_CMY-98_ and *qnrB12*. *C. cronae* ST141, isolated from fennec fox, contains *bla*_CMY-98_ (4). No mobile genetic elements were detected. Thus, our description of *C. cronae* ST880 strain Awk will facilitate genomic comparisons, with direct impact on public health surveillance of the *Citrobacter* genus.

### Data availability.

This whole-genome shotgun project has been deposited at DDBJ/ENA/GenBank under the accession number JAMCOT000000000.1 (BioProject accession number PRJNA836736 and BioSample accession number SAMN28171602). This is the first version of this genome. The raw sequence reads have been deposited in the Sequence Read Archive (SRA) under accession number SRR19520192.
